# Transcutaneous electrical acupoint stimulation improves endometrial receptivity resulting in improved IVF-ET pregnancy outcomes in older women: a multicenter, randomized, controlled clinical trial

**DOI:** 10.1186/s12958-022-00997-0

**Published:** 2022-08-22

**Authors:** Xiaojun Feng, Na Zhu, Shuo Yang, Li Wang, Wei Sun, Rong Li, Fei Gong, Songping Han, Rong Zhang, Jisheng Han

**Affiliations:** 1 Center of Reproductive Medicine, The Second Hospital Affiliated to Shandong University of Traditional Chinese Medicine, Jinan, 250001 China; 2 Reproductive & Genetic Hospital of CITIC-XIANGYA, Changsha, 410000 China; 3 Center of Reproductive Medicine, Department of Obstetrics and GynecologyKey Laboratory of Assisted Reproduction, Ministry of EducationBeijing Key Laboratory of Reproductive Endocrinology and Assisted Reproductive Technology, Peking University Third Hospital, Beijing, 100191 China; 4 Wuxi Shengpingxintai Medical Technology Co., Ltd, Wuxi, 214091 China; 5 Department of Neurobiology, School of Basic Medical Sciences, Key Lab for Neuroscience, Neuroscience Research Institute, Peking UniversityPeking University Health Science CenterThe Ministry of EducationThe Ministry of Health, Beijing, 100191 China; 6 Department of Integration of Chinese and Western Medicine, School of Basic Medical Sciences, Peking University, Beijing, 100191 China

**Keywords:** Infertility, Assisted reproduction technology, Acupuncture, TEAS, In vitro fertilization-embryo transfer, Clinical pregnancy, Endometrial receptivity, Pinopodes

## Abstract

**Objective:**

To examine the effects and mechanisms of transcutaneous electrical acupoint stimulation (TEAS) on pregnancy outcomes in women undergoing in vitro fertilization (IVF)-embryo transfer (ET).

**Design, setting, and participants:**

This efficacy study was a multicenter, randomized, controlled clinical trial (RCT) in women receiving IVF-ET. The mechanistic study was a single-center RCT.

**Interventions:**

The participants received TEAS vs. no TEAS treatment.

**Main outcome measures:**

In the efficacy study, the primary outcomes were the rates of clinical pregnancy, embryo implantation, and live birth. In the mechanistic study, sex hormones and endometrial protein expression were examined.

**Results:**

Ultimately, 739 participants were enrolled (367 and 372 in the TEAS and control groups, respectively). The clinical pregnancy rate was higher in the TEAS group than in the controls (55.1% vs. 46.7%, *P* = 0.03). There were no significant differences in embryo implantation, biochemical pregnancy, and live birth rates between the two groups (all *P* > 0.05) in the study population. In women > 35 years, the clinical pregnancy rates, embryo implantation rates and live birth rates in the TEAS and control groups were 48.9% vs. 23.7% (*P* = 0.004),30.8 vs. 13.9% (*P* = 0.001) and 34.0% vs. 19.7% (*P* = 0.06) respectively. In the mechanistic study with 120 participants, on the theoretical embryo implantation day, better developed endometrial pinopodes, elevated endometrial integrin α1β1/αVβ3, leukemia inhibitory factor, and elevated serum progesterone levels were found in the TEAS group compared with controls.

**Conclusion:**

TEAS significantly improved the clinical pregnancy rate in women undergoing IVF-ET, especially in women of older age. It might be due to improved endometrial receptivity.

**Trial registration:**

ChiCTR-TRC-13003950.

## Introduction

Infertility is the inability to conceive after 1 year of unprotected sexual intercourse [1–4]. Infertility occurs in approximately 15% of reproductive-aged couples worldwide and is more common in developing countries [2]. The causes of infertility in couples are multifactorial, including both male and female factors (40%), male factors alone (26%-30%), ovulation disorders (21%-25%), tubal factors (14%-20%), cervical, uterine, or peritoneal disorders (10%-13%), and idiopathic disorders (25%-28%) [1–4].

The treatment of infertility is based on the underlying causes. The likelihood of achieving a live birth through treatment for female infertility largely depends on the patient’s age. Assisted reproduction technology (ART) refers to all treatments or procedures to help women become pregnant. In vitro fertilization (IVF) involves oocyte extraction, fertilization in the laboratory, and embryo transfer (ET) into the uterus through the cervix [5]. IVF-ET can be performed on natural cycles, but the likelihood of achieving live birth is lower than when using controlled ovarian stimulation (COS) [6]. Nevertheless, the clinical pregnancy rate of women after IVF-ET remains low (30%-40%) in general [6–9].

Acupuncture has been suggested to increase female infertility and improve IVF-ET outcomes [10–13], but the results are sometimes controversial [14, 15]. There is a need for more high-quality randomized controlled trials to prove or disprove the efficacy of acupuncture in infertile women [10, 11]. Transcutaneous electrical acupoint stimulation (TEAS) is a noninvasive intervention derived from electroacupuncture as an alternative to manual or electroacupuncture [16, 17]. Like acupuncture, TEAS has been shown to have a wide range of indications, including pain relief, treatment of digestive disorders, prevention of nausea and vomiting, management of muscular ailments, management of body weight, and treatment of depression, anxiety, addiction, and stroke, as reviewed by Jun et al. [18]. TEAS has been shown to improve the implantation and pregnancy rates in patients with implantation failure [19] or recurrent implantation failure [20]. Our previous work demonstrated the benefits of TEAS in women undergoing IVF-ET [19–21]. Zhang et al. [21] showed that TEAS improved the outcomes of 309 women undergoing ET (either fresh or frozen embryos), with or without intracytoplasmic sperm injection (ICSI). Hsu et al. [19] showed that low-frequency TEAS improved implantation and clinical pregnancy rates. Shuai et al. [20] examined the effect of TEAS and found that the intervention improved the pregnancy outcomes in a study of 122 women with recurrent implantation failure. An expert consensus in China suggested various reproductive conditions in which TEAS could improve the outcomes [22].

The most important factors for pregnancy are the quality of the embryos and the maternal status. Few studies have explored the effect of acupuncture on endometrial receptivity. Obviously, embryo quality after oocyte retrieval and fertilization cannot be affected by acupuncture treatment. Therefore, it is likely that the maternal reproductive system and probably endometrial receptivity are the targets for acupuncture. We hypothesized that acupuncture increases endometrial receptivity by activating pregnancy-promoting cytokines such as integrin [23], leukemia inhibitory factor (LIF) [24], and heparin-binding EGF-like growth factor (HB-EGF) [25], thereby regulating the time window to facilitate the implantation.

This multicenter, randomized, controlled clinical trial examined the effects on clinical pregnancy, embryo implantation, abortion, and live birth rates of a 30-min, 2-Hz TEAS treatment given one day before and 30 min after ET in women undergoing IVF. This study also examined the association between cytokines and endogenous luteal function, serum levels of estradiol (E) and progesterone (P), and endometrial expression of pinopodes. The results might provide useful information for the mechanisms of action of TEAS alongside ART.

## Material and Methods

### Part 1. Efficacy study

#### Study design

This study was a multicenter, randomized, controlled clinical trial. It was approved by the Peking University Institutional Review Board (Approval No. IRB00001052-13,011, IRB00001052-10,060) and registered at the Chinese Clinical Trial Registry, a World Health Organization International Clinical Trial Registration Platform (http://www.chictr.org.cn) (#ChiCTR-TRC-13003950). All participants signed the informed consent form before any study procedure was undertaken.

#### Participants

Eligible women who received IVF-ET at the Second Affiliated Hospital of Shandong University of Traditional Chinese Medicine, Reproductive & Genetic Hospital of Citic-Xiangya, and Peking University Third Hospital between December 2013 and December 2016 were enrolled. The inclusion criteria were 1) indications for IVF-ET, as described in the Ministry of Health Notification on the Revision of Technique Standards, Basic Standards, and Ethical Principles of Human Assisted Reproductive Technology and Human Sperm Bank (Weikejiaofa [2003] 176) [which were i) gamete transport disorders in women due to many causes, ii) ovulation dysfunction, iii) endometriosis, iv) immunological infertility, or v) unknown infertility], 2) 25–45 years of age, 3) body mass index (BMI) of 18–31 kg/m^2^, 4) normal ovarian functions, and 5) fresh or cryopreserved embryos. The exclusion criteria were 1) with indications for IVF-ET, but underwent IVF/ICSI due to male factor infertility, 2) with organic disorders such as hydrosalpinx or intrauterine adhesion, 3) with contraindications to ART [i) the male or female of the infertile couple had severe psychological disorders, acute genitourinary system infection, or sexually transmitted disease, ii) with hereditary diseases that should not consider pregnancy according to the Chinese Maternal and Infant Healthcare Law, i.e., that could not be diagnosed by a prenatal genetic test, iii) male or female of the infertile couple was with addictions to drugs or alcohol, iv) male or female of the infertile couple had a history of exposure to teratogenic doses of radiations, drugs, or toxins/pollutants and was still in the effect period; or v) urine human chorionic gonadotropin (hCG) test showed positive results (already pregnant)], or 4) with contraindications to TEAS [i) implanted with a pacemaker or other electronic medical equipment, ii) concomitant extracorporeal membrane oxygenation (ECMO), electrocardiogram (ECG), or microwave therapy apparatus, and iii) skin lesions at the target acupoints].

#### Randomization and blinding

A central randomization system was used. A random sequence was generated by a computer, and the participants were randomized sequentially by enrollment order. When a patient was considered eligible by an investigator, the basic information of the patient was transferred to the central randomization system through the internet, and then the treatment strategy for each subject was allocated: TEAS or control (no TEAS).

Each participant and sample was assigned a unique number. All tests were performed by examiners blind to the grouping of the participants. All data were analyzed by third-party statisticians who were not part of the study staff. The participants could not be blinded to the treatments.

#### Intervention

**Fig. 1:**
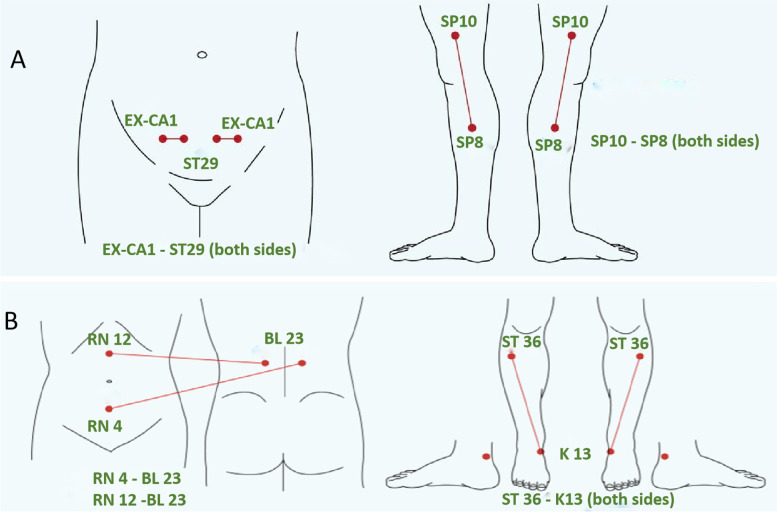
Illustration of acupoints used in TEAS intervention. **A** Acupoints at 24 h before embryo transfer. **B** Acupoints at 30 min after embryo transfer

Prior to IVF, the comorbidities were managed (e.g., patients with hypothyroidism received treatment to normalize the TSH levels). Patients with definite uterine lesions underwent treatment (e.g., endometrial polyp ablation). For the women in the TEAS group, electrical acupoint stimulation via skin pads with a frequency of 2 Hz was applied for 30 min at 24 h before ET and 30 min after ET. All physicians responsible for the acupoint electrical stimulation procedure received group training and had a background related to TCM. The acupoints were selected according to the principles of TCM gynecology. The acupoints selected for stimulation 24 h before ET (Fig. 1A) were bilateral Diji (SP8), Guilai (ST29), Zigong (NR19), and Xuehai (SP10). The stimulation intensity was set to 10 mA, which was twofold the sensory threshold (~ 5 mA). The current was increased to 15–20 mA if the woman could tolerate it and feel comfortable. The acupoints selected for stimulation at 30 min after ET (Fig. 1B) were bilateral Zusanli (ST36), Taixi (KI3), Shenyu (BL23), Guanyuan (RN4), and Zhongwan (NR12). The current was initially set to 10 mA and gradually increased according to the tolerability of the woman to electrical stimulation. The stimulation intensity used in each treatment was recorded.

All participants received standard care according to the IVF-ET protocol. The women in the control group did not receive any TEAS treatment.

#### IVF-ET Protocol

For women receiving fresh ET, routine luteal support was started on the day of oocyte retrieval. For women receiving cryopreserved ET, routine luteal support was started 3 days before transfer. Two D3 embryos were transferred to each participant. The blood hCG was tested for biochemical pregnancy 14 days after embryo transfer, and the drug therapy was continued until the 12^th^ gestational week for pregnant women.

#### Outcomes

The study’s primary outcomes were the clinical pregnancy rate (number of women with clinical pregnancy/total number of women), the embryo implantation rate (number of implanted embryos/total number of embryos transferred), and the live birth rate (number of live births/total number of women). The secondary outcomes included the biochemical pregnancy rate (number of women with biochemical pregnancy/total number of women), the abortion rate (number of abortions/number of clinical pregnancies), and the rate of birth defects.

The pregnancy outcome was followed by telephone calls if the woman chose to deliver the baby to another hospital. The woman was requested to report the pregnancy outcomes by providing the testing results, including blood hCG examination reports and ultrasound examination reports.

Biochemical pregnancy was defined as a positive urine hCG test 14 days after ET. Clinical pregnancy was defined as ultrasound showing a gestational sac, fetal bud, and fetal heartbeat 45 days after ET. The number of implanted embryos was the number of live embryos. Early abortion was defined as embryo loss within 12 weeks. Live birth was defined as neonates living more than 24 weeks after birth. Birth defects were recognizable structural or functional defects at birth which was formed in utero (i.e., excluding birth injuries).

#### Safety evaluation

Intervention-related adverse events (AEs), including but not limited to a small amount of brown vaginal discharge, abdominal distension, slight pain in the left lower abdomen, and pain in the waist and abdomen, were recorded by follow-up through telephone calls or hospital visits.

#### Sample size calculation

A superiority study design was used in this study. The clinical pregnancy rate was selected as the primary treatment efficacy indicator. According to preliminary study results [21], the clinical pregnancy rate in the control group was presumed to be 40%, and the clinical pregnancy rate in the study group was presumed to be 50%. The sample size of the study and control groups was determined to be 385 when the one-sided α was 0.025, the study power (1-β) was 80%, and the ratio of participants in the study group to the control group was 1:1. After drop-off, exclusion (10%) and central bias (blind analysis of the data in each study center when 770 participants were included) showed that the mean pregnancy rate at the Reproductive & Genetic Hospital of Citic-Xiangya was 60.5%, which was higher than at the other two hospitals, at 40.4% and 38.6%, respectively. Further analysis showed that the mean age of the women enrolled at the Reproductive & Genetic Hospital of Citic-Xiangya was 30 years, younger than at the other two hospitals (32 and 33.5 years, respectively). When age differences were considered, the sample size in each group was determined to be 483.

### Part 2. Mechanistic study on endometrial receptivity

#### Participants and design

A group of 120 women preparing to receive frozen cryopreservation embryo transplantation was recruited. They were all from the Second Affiliated Hospital of Shandong University of Traditional Chinese Medicine and were on the waiting list. The participants were randomly assigned into two groups: the control and TEAS groups. The patients in the TEAS group were treated on days 16 and 17 of the menstrual cycle in the same way described in the “Intervention” section.

#### Peri-implantation endometrium samples

Mild curettage promotes endometrial growth and helps analyze the causes of ET failure. Women eligible for cervical dilatation and curettage underwent curettage after signing the informed consent (approval No. IRB00001052-10,060). Endometrial biopsy specimens were washed immediately in normal saline and divided into two parts: one was immediately frozen at -80 °C for the assessment of integrin, LIF, and HB-EGF. The other part was fixed in 0.1 M phosphate buffer (pH 7.4) containing 3% glutaraldehyde for scanning electron microscopy analysis.

#### Enzyme immunoassay for endometrial Integrin α1β1, α4β1, α_V_β_3_, HB-EGF, and LIF

A total of 110 endometrial samples were used to measure the levels of immunoreactive cytokines by the double antibody sandwich ABC-ELISA method (Shanghai Xitang Biotechnology Company Limited), including integrin α1β1, α4β1, αVβ3, HB-EGF, and LIF. The assays were performed according to the manufacturer’s instructions. The ABC-ELISA was highly sensitive, and the detection limits were 15 ng/ml for integrin α1β1 and α4β1, 15 pg/ml for integrin αVβ3, 30 pg/ml for LIF, and 16 pg/ml for HB-EGF) with very low cross-reactivity with other molecules. The R-values of the standard curves were > 0.999 for all assays.

#### Scanning electron microscopy

Thirty-nine endometrial samples (20 in the control group and 19 in the TEAS group) were used to perform electron microscopy scanning. The endometrial biopsy samples were fixed in 2.5% glutaraldehyde in 0.1 M phosphate-buffered saline (PBS, pH 7.4), rinsed in Sorensen’s buffer, postfixed in 2% osmium tetroxide for 1 h, rinsed three times in Sorensen’s buffer for 10 min, dehydrated in graded alcohols, dried in a critical point drier using carbon dioxide, coated with palladium-gold, and examined using a scanning electron microscope. Depending on the size and epithelium presentation, 5 to 15 scans were performed for each sample. The expression of pinopodes was assessed in two ways: 1) the ratio of pinopode area to scanning area (a ratio of < 20% was ranked low, 20%-50% as moderate, and > 50% as high) and 2) the number of pinopodes in one high-power field (area of 3019.6 µm^2^).

#### Blood sampling and assessment of serum E and P

The 39 patients whose endometrium was subjected to electron microscopy scanning were also subjected to the assessment of serum E and P. The collection of blood samples took place on the morning of day 21 of the menstrual cycle (implantation window phase), with the patients previously informed to fast overnight. Venous blood (2 mL) was collected into tubes, placed at room temperature for 30 min, and centrifuged at 1600 × g for 15 min at 4 °C. The serum was isolated, divided into 500-μL aliquots, and immediately stored at -80 °C. E and P levels were measured using the original auxiliary reagents for the Roche electrochemiluminescence immunoassay system Cobas E601 (Hoffmann-La Roche, Ltd, Inc., Switzerland). The analyses were conducted blindly regarding which group the samples belonged to. For each assay, all samples were run at the same time.

#### Statistical analysis

Data were managed and analyzed using SAS 9.1.3 (SAS Institute, Cary, NY, USA). Continuous data were tested using the Kolmogorov–Smirnov test. Those with a normal distribution were presented as the means ± standard deviations and tested with Student’s *t*-test; otherwise, they were presented as medians (ranges) and tested using Wilcoxon’s rank-sum test. Categorical data are presented as frequencies and percentages and were analyzed using the chi-square test or Fisher’s exact test. Wilcoxon’s rank-sum test or the CMH test was performed to analyze ordinal data. An unpaired *t*-test was used to compare the changes in serum E and P, pinopode expression, endometrial integrin α1β1, α4β1, αVβ3, HB-EGF, and LIF levels between the two groups. Linear regression analysis was used to detect the correlation of the plasma levels of P with endometrium integrin α1β1, α4β1, and αVβ3, HB-EGF, and LIF levels and pinopode expression. All statistical analyses were two-sided, and *P* < 0.05 was considered statistically significant (unless otherwise described).

## Results

### Characteristics of the participants

**Fig. 2:**
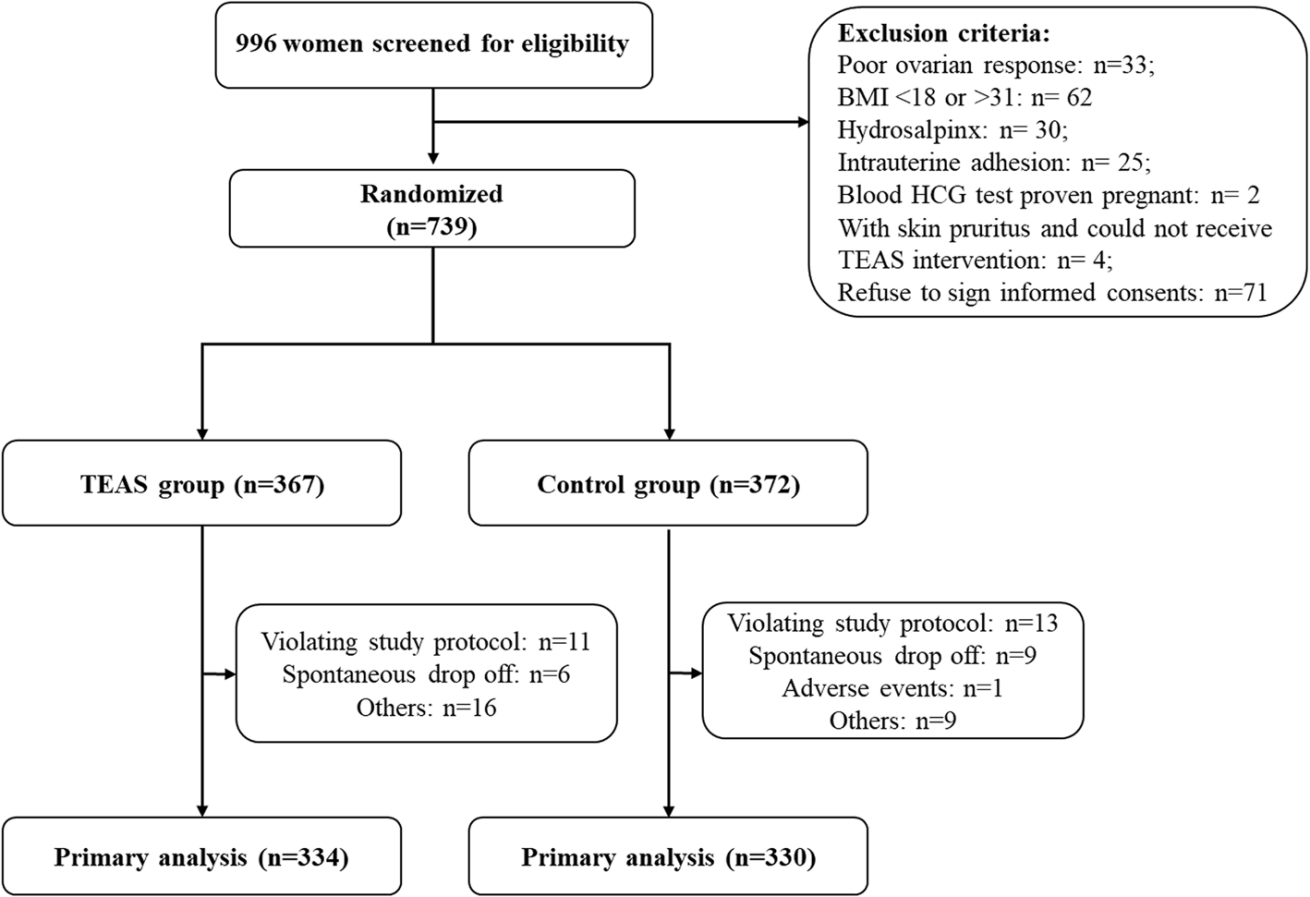
Participant flowchart. BMI: body mass index; hCG: human chorionic gonadotropin; TEAS: transcutaneous electrical acupoint stimulation

Figure 2 presents the enrollment process: 966 women were screened, of which 156 did not meet the inclusion criteria (poor ovarian response in 33 women, BMI < 18 or > 31 kg/m^2^ in 62 women, hydrosalpinx in 30 women, intrauterine adhesion in 25 women, positive blood hCG test in two women, and skin pruritus of the areas for TEAS treatment in four women) and 71 refused to participate. Therefore, 739 patients were enrolled in the study, of which 367 were in the TEAS group and 372 were in the control group. Five women in the TEAS group and three in the control group were excluded due to missing data. The baseline characteristics of the participants are shown in Table 1. There were no significant differences between the two groups for any characteristics.

**Table 1:** Characteristics of the participants

Characteristics	TEAS group (*n* = 362)	Control group (*n* = 369)	P
Age, years (mean ± SD)	31.5 ± 4.4	31.7 ± 4.7	0.749
Race, n (%)			0.317
Han	345 (95.3)	357 (96.8)	
Minorities	17 (4.7)	12 (3.3)	
BMI, kg/m^2^ (mean ± SD)	22.5 ± 3.0	22.4 ± 3.1	0.556
Drinking history, n (%)	360 (99.5)	368 (99.7)	0.621
Years of infertility (years)	3 (2,6)	4 (2,6)	0.579
History of acupuncture, n (%)	15 (4.1)	10 (2.7)	0.289
Times of IVF-ET, n (%)			0.423
1	185 (51.1)	196 (53.1)	
2–3	175 (48.3)	168 (45.5)	
≥ 4	2 (0.6)	5 (1.4)	
Causes of infertility, n (%)			0.824
Gamete transport disorders	333 (92.0)	341 (92.4)	
Ovulation dysfunction	18 (5.0)	14 (3.8)	
Endometriosis	6 (1.7)	8 (2.2)	
Immunological infertility	5 (1.4)	6 (1.6)	
Embryo, n (%)			0.771
Fresh	183 (50.6)	190 (51.6)	
Cryopreserved	179 (49.5)	178 (48.4)	
Comorbidities (before treatment), n (%)			0.953
Hyperlipemia	2 (0.6)	0	
Hyperprolactinemia	2 (0.6)	3 (0.8)	
Hyperthyroidism	1 (0.3)	1 (0.3)	
Subhypothyroidism	2 (0.6)	2 (0.5)	
Hypothyroidism	3 (0.8)	1 (0.3)	
Uterine cavity lesions, n (%)			0.833
Submucous myoma	8 (2.2)	11 (3.0)	
Uterine mediastinum	2 (0.6)	0	
Thin endometrium due to repeated curettages	1 (0.3)	3 (0.8)	
Drugs, n (%)			
Combined drug therapy after inclusion	122 (33.7)	125 (33.9)	0.938
Continue drug therapy after inclusion	24 (6.6)	34 (9.2)	0.219

*TEAS* transcutaneous electrical acupoint stimulation, *BMI* body mass index, *IVF-ET* in vitro fertilization-embryo transfer

### Pregnancy outcomes

The initial analysis shows that the clinical pregnancy rate was higher in the TEAS group than in the control group (55.1% vs. 46.7%, *P* = 0.03), but there were no statistically significant differences between the two groups regarding the embryo implantation rate (37.1% vs. 33.9%, *P* = 0.252), biochemical pregnancy rate (57.2% vs. 52.7%, *P* = 0.248), live birth rate (44.0% vs. 40.0%, *P* = 0.295), early abortion rate (19.6% vs. 12.3%, *P* = 0.078), late abortion rate (2.6% vs. 3.0%, *P* > 0.999), total abortion rate (21.7% vs, 14.9%, *P* = 0.072), or birth defect rate (1.0% vs. 1.0%, *P* > 0.999) (Table 2).

**Table 2:** Primary and secondary outcomes

Outcomes, n/N (%)	TEAS group	Control group	P
Primary outcomes			
Clinical pregnancy rate	184/334 (55.1)	154/330 (46.7)	0.030
Embryo implantation rate	226/609 (37.1)	200/590 (33.9)	0.252
Live birth rate	147/334 (44.0)	132/330 (40.0)	0.295
Secondary outcomes			
Biochemical pregnancy rate	191/334 (57.2)	174/330 (52.7)	0.248
Abortion rate			
Early abortion rate	36/184 (19.6)	19/154 (12.3)	0.078
Late abortion rate	4/151 (2.7)	4/135 (3.0)	> 0.999
Total abortion rate	40/184 (21.7)	23/154 (14.9)	0.072
Birth defects rate	2/187 (1.1)	2/174 (1.1)	> 0.999

*N* number, *N* total number, *TEAS* transcutaneous electrical acupoint stimulation

**Fig. 3:**
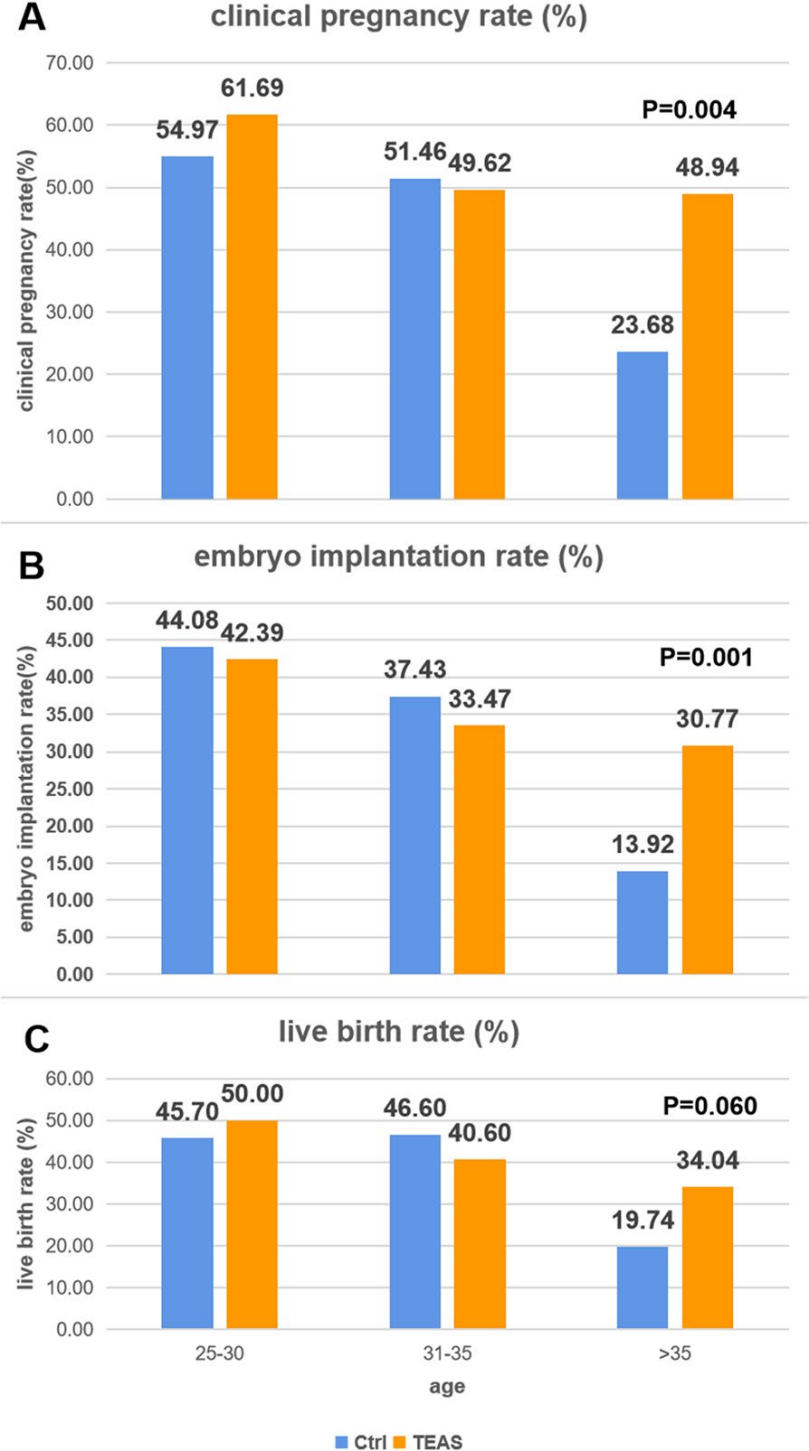
Comparison of pregnancy outcomes **A** clinical pregnancy rate, **B** embryo implantation rate, and **C** live birth rate in different age groups

The stratified analysis for the different age groups was carried out considering that younger and older women might have different causes of infertility and hence different responses to TEAS. The stratified analysis showed that TEAS did not affect any primary or secondary pregnancy outcomes examined in women between 25 and 30 years or between 31 and 35 years. In contrast, the therapeutic effects observed in women over 35 years were rather striking: the clinical pregnancy rates in the TEAS and control groups were 48.9% vs. 23.7% (*P* = 0.004), the embryo implantation rates were 30.8% vs. 13.9% (*P* = 0.001), and the live birth rates were 34.0% vs. 19.7% (*P* = 0.06) (Fig. 3). Hence, TEAS therapy appears to be more effective in women over 35 years, although the general success rate for IVF-ET was lower in this older population.

### Safety

Table 3 shows that TEAS had no detrimental AE profile compared with the control group. The occurrence of AEs was 0.3% in the TEAS group and 0.9% in the control group.

**Table 3:** Adverse events related to treatments

Adverse events, n (%)	TEAS group (*n* = 334)	Control group (*n* = 330)
Small amount of brown vaginal discharge	1 (0.3)	0
Abdominal distension	0	1 (0.3)
Slight pain in left lower abdomen	0	1 (0.3)
Pain in waist and abdomen	0	1 (0.3)

*TEAS* transcutaneous electrical acupoint stimulation

### Endometrial receptivity

**Fig. 4:**
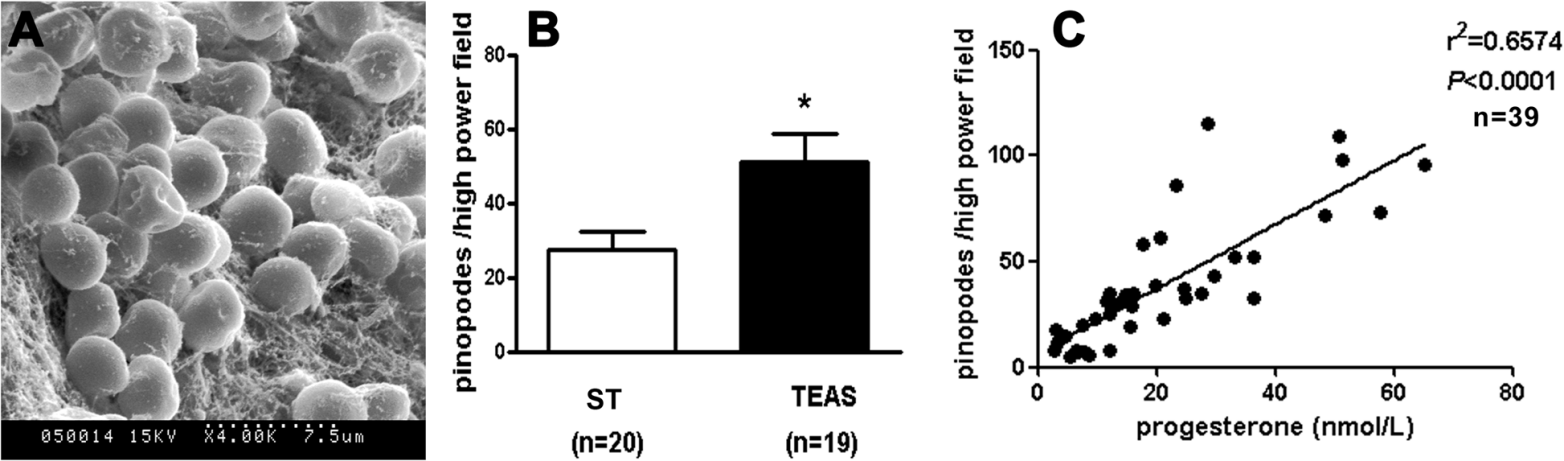
Influence of TEAS on endometrial pinopode development in women on the theoretical embryo implantation day. Photograph **A** showing pinopodes in scanning electron microscopy. Histogram **B** showing that the mean number of pinopodes seen per high-power field was higher in the TEAS group than in the control group (*P < 0.05, two-tailed Student’s t-test). Linear regression analysis **C** showed that the serum progesterone levels were positively correlated with the number of endometrial pinopodes

A total of 120 women were randomly and equally divided into two groups: the control and TEAS groups. They were all preparing to receive frozen-cryopreserved embryo transplants. After TEAS treatment on days 16 and 17 of the menstrual cycle (the control group received no treatment), endometrial samples were collected on day 21 to detect cytokines, and 39 samples (20 in the blank group and 19 in the 2 Hz TEAS group) were used for scanning electron microscopy. Scanning electron microscopy revealed that pinopodes appeared in the endometrial samples on day 21 of the menstrual cycle, but the density differed among participants. According to the area ratio of pinopodes (Fig. 4A) in a high-power field, the participants were divided into the low (< 20%), moderate (20%-50%), and high (> 50%) groups. The results showed that the density of pinopodes was higher in the TEAS group. The mean number of pinopodes per high-power field was higher in the TEAS group than in the control group (51.2 ± 7.8 vs. 27.5 ± 4.9, *P* = 0.011) (Fig. 4B). A positive correlation was also found between serum P level and the density of pinopodes (*P* < 0.001) (Fig. 4C). No such correlation was found between serum E levels and the abovementioned cytokines and pinopode expression (data not shown).

**Fig. 5:**
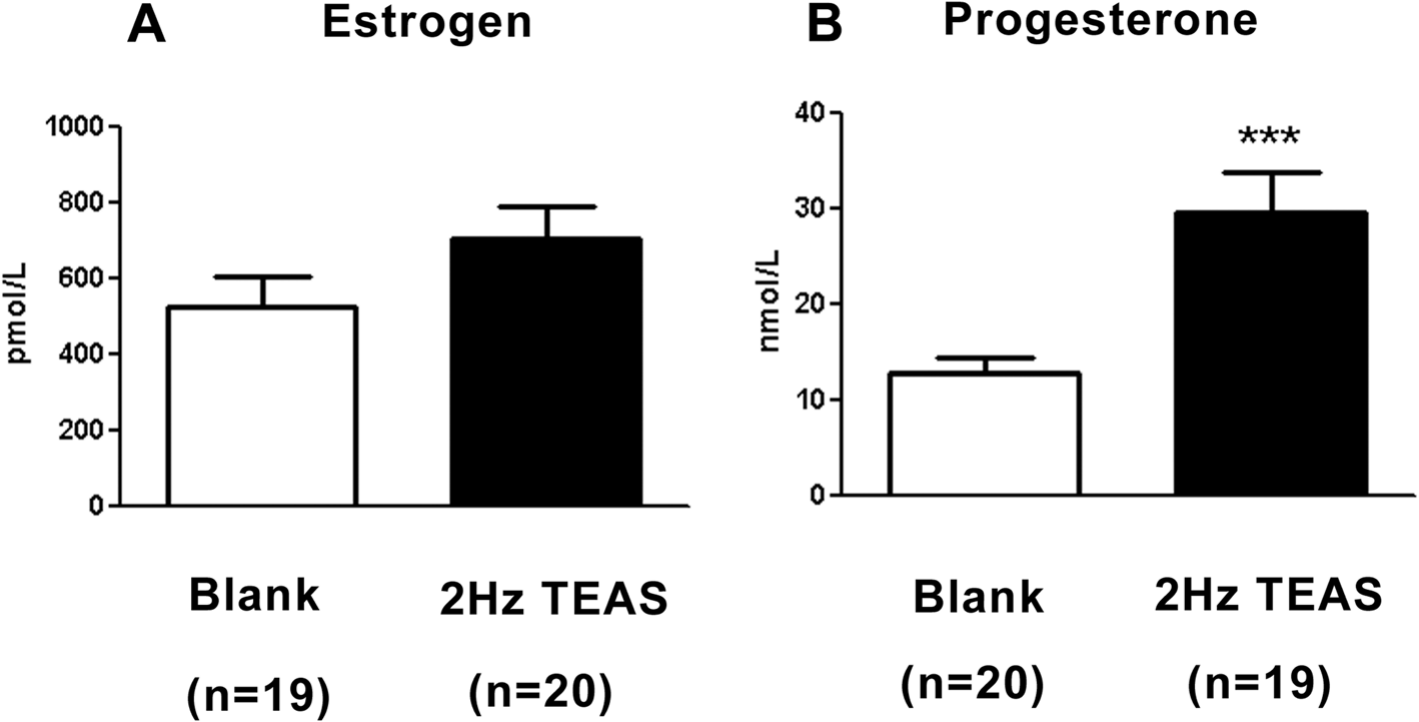
Changes in serum sex hormones of women on the theoretical implantation day. Histograms showing serum estrogen **A** and progesterone **B** levels 3 days after TEAS intervention. The TEAS group had increased serum levels of progesterone but not estrogen. Data are expressed as the mean ± S.E., with ***P < 0.001 compared with the control group (unpaired two-tailed Student’s t-test)

**Fig. 6:**
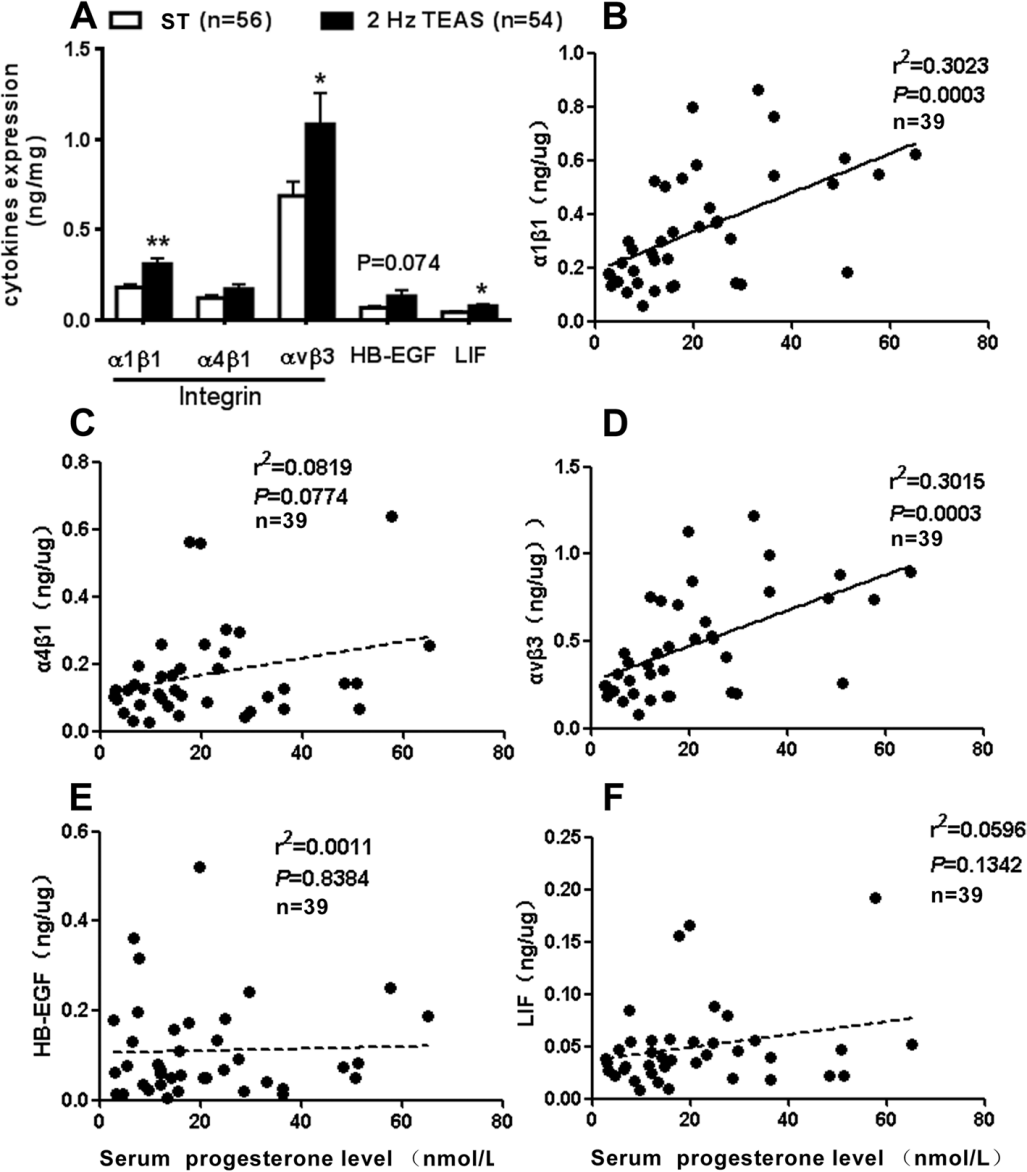
Changes in integrin cytokines in the endometrium of women on the theoretical embryo implantation day and the correlation between endometrial cytokine levels and serum progesterone levels. TEAS increased integrin α1β1 and αVβ3 but not α4β1 in the endometrium (A). Linear regression analysis showing a positive correlation with endometrial content of integrinα 1β1 (B) and αVβ3 (D) but not α4β1 (C), HB-EGF E or LIF (F) levels. Data are expressed as the mean ± S.E., with *P < 0.05 and **P < 0.01 representing statistically significant differences compared with the control group (unpaired two-tailed Student’s t-test)

The serum endogenous P level was significantly higher in the TEAS group than in the control group [29.57 ± 4.36 vs. 12.80 ± 1.56, *P* < 0.001] (Fig. 5 B). No such difference was found in serum E levels (Fig. 5 A). In addition, the TEAS group showed higher endometrial contents of integrin α1β1 (0.31 ± 0.03 vs. 0.18 ± 0.02, *P* < 0.001), integrin αVβ3 (1.08 ± 0.17 vs. 0.69 ± 0.08, *P* = 0.037) (Fig. 6A), and LIF (0.07 ± 0.01 vs. 0.04 ± 0.01, *P* = 0.012). No such changes were observed in endometrial integrin α4β1 (0.17 ± 0.02 vs. 0.12 ± 0.02, *P* = 0.123) or HB-EGF (0.13 ± 0.03 vs. 0.07 ± 0.01, *P* = 0.074) (Fig. 6A).

The correlation analysis (Fig. 6B-F) revealed that in all 39 patients, the serum P levels showed a positive correlation with endometrial contents of integrin α1β1 (*P* < 0.001) (Fig. 6B) and αVβ3 (*P* < 0.001) (Fig. 6D) but not with integrin α4β1 (*P* = 0.077) (Fig. 6C), HB-EGF (*P* = 0.838) (Fig. 6E), or LIF (*P* = 0.134) (Fig. 6F).

## Discussion

TEAS is a noninvasive technique derived from acupuncture and electroacupuncture. TEAS involves no needles and has a safer profile than acupuncture. The standard procedure requires less training for physicians and can be more readily accepted by the patients [16, 17]. It is effective in the management of various chronic conditions [18]. TEAS has been suggested to improve the outcomes of IVF-ET [19–21], but the quality of the evidence remains low, largely due to the small sample sizes. Therefore, this multicenter randomized controlled trial with a large sample size was designed to examine the effects of TEAS on pregnancy outcomes.

Age is a major factor affecting fertility and the outcomes of IVF-ET. Advanced maternal age is associated with poorer outcomes than younger women [26–28]. The initial analysis showed that TEAS only modestly improved the clinical pregnancy rate, and none of the other pregnancy outcomes was significantly altered when all women from 25 to 45 years old were included in the analysis. On the other hand, the stratified analysis revealed a different picture when the patients were stratified according to their age. There were large and clinically significant improvements in pregnancy outcomes in women over 35. There was a strong trend for improving the live birth rate in this group of older women. The lack of statistical significance in the live birth rate is likely due to the smaller sample size and lower statistical power because of stratification. It seems likely that the moderate increase in the clinical pregnancy rate and no effect on other outcomes seen in the whole patient population without stratification were due to the inclusion of younger patients. These patients had relatively normal endometrial receptivity and thus were non-responders for TEAS. The dilution of the therapeutic effects might also be responsible for the smaller effects reported in previous studies [16, 17, 19–22, 29]. It is well established that the incidence of diminished ovarian function and declining endometrial receptivity increase with the increasing age of women and become more critical to infertility [30]. Mechanistically, acupuncture and TEAS have been suggested to improve the function of the hypothalamo-pituitary-ovarian axis and prevent premature ovarian failure [29]. For example, TEAS increased antral follicle count and AMH levels and decreased FSH and circulating ratio of LH/FSH [29], increasing endometrial thickness [20, 31, 32]. An endometrium of at least 7 mm has been deemed necessary for gestation [33]. Other mechanisms include increased ovary and endometrial focal blood flow [34–36] and modulation of chemokines, integrins, and growth factors for maintaining normal endometrial receptivity [24, 37]. These may be the major mechanisms for the therapeutic effects of TEAS and are the major deficiency in women over 35 with infertility. In contrast, these might not be the major causes of infertility in young women. Hence, the results suggest that TEAS could be an alternative in managing older women with infertility, a population of infertile patients with more complicated management and poorer outcomes [26–28].

In this study, TEAS appeared to be safe, and no adverse safety profile was reported on reproductive functions. These results are consistent with previous studies of TEAS and acupuncture in general [10–15, 19–21]. It is not clear why there was a trend for a higher abortion rate in the treatment group, especially in women over 35. It is possible that poor embryo quality, ovarian dysfunction, and abnormal endometrium receptivity were the major causes of infertility in women over 35. On the other hand, the intervention partially improved receptivity but was not designed to optimize pregnancy. Extra implanted embryos faced a higher risk, and some of them were lost in early pregnancy (12 weeks after ET). Still, it is true only if the increased miscarriage rate is equal to or less than the increased pregnancy rate. Therefore, future studies should compare older patients among themselves. These conditions were more prominent in older women and were associated with a higher early abortion rate. There was no difference between the two groups in the late abortion rate (Table 2). It seems important to expand the study to find ways to maintain extra implanted embryos because of TEAS treatment. The results of our previous trial with prolonged treatment (3 months) of TEAS showed that TEAS improved the quality of embryos [29], which might help reduce the early abortion rate in women over 35 years.

The most exciting findings in this study are the effects of TEAS on endometrial receptivity, which might be the direct cause of increased clinical pregnancy and implantation rates. It is the first study demonstrating that the therapeutic effect of acupuncture-related techniques on pregnancy outcomes is due to an elevation of pregnancy-promoting cytokines in human studies. In a previous study, Zhai et al. [38] randomized 200 patients into five groups (mock TEAS and four different current intensity of TEAS, once a day on days 10–13) and examined endometrial thickness, number of ovarian follicles, number of harvested oocytes, mature oocyte rate, rate of high-frequency embryos, and clinical pregnancy rate. They showed that TEAS could improve the ovarian state and that TEAS at ≥ 40 mA could improve the endometrial state and receptivity and oocyte retrieval [38]. Hsu et al. [19] found that TEAS was a promising technique to improve reproductive outcomes in difficult cases of IVF-ET, but little research has shown how it works.

As supported by Zhai et al. [38], pinopode expression was higher after TEAS stimulation, suggesting that the endometrium was more ready to accept the embryo than the control group. In addition, TEAS increased the P but not E levels in the serum. During receptivity, hairy-like epithelial cell microvilli transiently fuse to a single flower-like membrane projection called the ‘pinopode’. Animal research showed that the number of pinopodes in the EA group was abundant [39], supporting the present study. In addition, significantly higher expression levels of pinopode-related markers, including integrin αvβ3, homeobox A10 (HOXA10), heparin-binding EGF-like growth factor (HBEGF), estrogen receptor alpha (ER*α*), and progesterone receptor (PR), were observed [39]. The higher expression of pinopodes could increase the contact area between a blastocyst and the endometrial surface, and pinopodes are regarded as classic biomarkers of the window of implantation in the human endometrial epithelium [40].

At the molecular level, integrin αVβ3 and HBEGF are associated with embryo implantation [41]. Several molecular markers are involved in pinopode formation, such as integrin, LIF, and HB-EGF [41]. LIF is one of the most important cytokines influencing endometrial receptivity during the early implantation window [42]. In the integrin family, αVβ3 has drawn more attention because its high expression correlates with the appearance of pinopodes, which is also a characteristic biomarker for endometrial receptivity [43].

In the present study, pinopode formation was closely associated with the serum P levels but not with E. Because it is difficult to obtain endometrial epithelium from women who received fresh embryo implantation, we chose women to receive frozen cryopreservation embryo transplantation. They did not receive any gonadotropin-releasing hormone or P in the harvest menstrual cycle, so the endogenous changes induced by TEAS were not affected by exogenous hormones. The results showed that an increase in P but not E appeared on the theoretical embryo implantation day. The molecular assessment revealed that TEAS increased the expression of integrin α1β1 and αVβ3 and LIF in the endometrium. A positive correlation between serum P levels and the levels of endometrial integrin α1β1 and αVβ3 suggested a possible causal relationship. In addition, higher pinopode expression was found in subjects with higher serum P. Compared with P, E did not seem to play a significant role in mediating the therapeutic effects of TEAS. Some animal experiments proved that acupuncture promotes the expression of endometrial LIF during the implantation period [39].

This study has some limitations. First, a sham or mock TEAS group was not used in this study due to the clear and strong sensation of TEAS, and the placebo effect could not be excluded. Consequently, blinding the participants could not be performed, introducing bias from participant factors even though the outcomes were objective. Second, the study was not designed to examine the effect of TEAS on the different causes of infertility. There is a possibility that different types of infertility respond differently to TEAS. Third, successful implantation was a complex process. Finally, as our final focus was on clinical pregnancy rate and embryo implantation rate, frozen embryo and fresh embryo transfer cycles were included, and the detailed IVF cycle characteristic was not collected during data entry.

## Conclusions

In conclusion, this randomized controlled trial of 731 women showed that TEAS improved pregnancy outcomes in women over 35 years of age and undergoing IVF-ET. There is no safety issue associated with TEAS. The mechanism of the therapeutic effect of TEAS might be related to increased corpus luteum support and endometrial receptivity to fertilized embryos.

## Data Availability

The datasets used and/or analyzed during the current study are available from the corresponding author on reasonable request.
